# Editorial: Machine learning and deep learning in biomedical signal analysis

**DOI:** 10.3389/fnhum.2023.1183840

**Published:** 2023-05-04

**Authors:** Bao Feng, Tianyou Yu, Hongtao Wang, Ke Liu, Wei Wu, Wansheng Long

**Affiliations:** ^1^Laboratory of Artificial Intelligence of Biomedicine, Guilin University of Aerospace Technology, Guilin, China; ^2^Center for Brain Computer Interfaces and Brain Information Processing, School of Automation Science and Engineering, South China University of Technology, Guangzhou, Guangdong, China; ^3^Faculty of Intelligent Manufacturing, Wuyi University, Jiangmen, China; ^4^College of Computer Science and Technology, Chongqing University of Posts and Telecommunications, Chongqing, China; ^5^Alto Neuroscience Inc., Palo Alto, CA, United States; ^6^Department of Radiology, Jiangmen Central Hospital, Jiangmen, China

**Keywords:** EEG based brain-computer interfacing, radiological imaging, deep learning—artificial neural network, automated parameterization, explainable learning, transfer learning, federal learning, domain adaptation

The electroencephalogram (EEG), based on brain–computer interfacing (BCI) and medical images generated as secondary or tertiary byproducts within neural activities, is widely used in clinical diagnosis, patient monitoring, and biomedical research. Therefore, how to effectively detect, analyze, and study biomedical signals is of great significance for human beings to study life phenomena and medical science. Recently, machine learning technologies, especially deep learning, have significantly advanced biomedical signal analysis. Following this line, the articles contemplated in this Research Topic show that this field of knowledge is rapidly expanding, and considerable advances have been made in the last few years. There remain various unsolved problems with respect to the use of advanced deep learning methods and machine learning in biomedical signal analysis, e.g., weak generalization, inexplicability, limited datasets, and data silos. In this Research Topic, we have an example of using a multi-modal deep learning model for the EEG or medical images, but the feasibility of these multi-modal works relies on raw datasets. Furthermore, the potential of transfer learning frameworks and adversarial learning is being studied in EEG or medical image processing.

Resting on their strong fitting ability for mass data, machine learning and deep learning models are popularly used to analyze biomedical signals such as the EEG and medical images, which have shown superiority in P300-BCI, motor imagery, and medical image processing, but their ability of generalization in small datasets is still limited. Research works devoted to studying proper machine learning and deep learning solutions of biomedical signals analysis, and the interpretability of models, are therefore of utmost importance. In this editorial, we summarize the main findings and viewpoints detailed within each of the seven contributing articles using deep learning in the EGG or medical image analysis.

The advantages of machine learning methods in decoding EGG signals have been discussed, and a sparse spatial decoding method based on the support vector machine has been proposed by Hou et al.. They found that the multi-modal dual-level stimulation paradigm is a powerful tool for enhancing the performance of motor imagery classification, and the proposed method can precisely select a few key frequency sub-bands from the filter bank.

Multi-modal biomedical signals can provide complementary physiological activity information so as to provide a more comprehensive and accurate interpretation of the brain function. For improving the performance of classification in motor imagery and mental arithmetic tasks, Qiu et al. proposed a multi-modal fusion framework to achieve the complementary characteristics between the EGG and functional near-infrared spectroscopy (fNIRS) based on multi-level progressive learning with multi-domain features. The experimental results prove that the task-related brain electrical and hemodynamic information can be fully extracted through multi-domain features, and the redundant information can be eliminated through the ASO algorithm. The multi-domain features of the EEG and fNIRS can be effectively fused through multi-level progressive machine learning. Zhou et al. reported that the modality-specific features extracted by deep networks are also important in aggregating task-related and complementary information from different modalities. Moreover, the ablation study proved that the bilateral symmetry of head in MRI images may help to progress the performance (Zhou et al.).

For the weak generalization and unsatisfying performance caused by the small amount of biomedical signal datasets, transfer learning is a popular and potential solution. Xuan et al. directly transferred natural image knowledge into a model of MRI image segmentation and achieved better accuracy than physicians. In this line, Chen and Chen proposed an adaptive selection-based dual-source domain feature matching network that can sift helpful knowledge into the target model from dual-source models. In addition to selecting beneficial knowledge from the source domain, mapping the source domain and target domain into a specific feature space with the same distribution is also a proper way. This mapping was performed by Liu and Cui. The utilization of the maximum mean discrepancy is also reported in this Research Topic. On the other hand, adversarial learning is a novel strategy to augment small datasets or improve the ability of generalization. Zhang et al. studied boundary and mask adversarial learning to aggregate helpful features of boundaries and assist the mask segmentation task.

The application of machine learning and deep learning is expanding in the field of biomedical signal analysis. The support vector machine-based, multi-modal, transfer, and adversarial learning works summarized here are practical and outstanding. These works are new, meaningful examples of the application of machine learning and deep learning in biomedical signal analysis, and more excellent works about this promising area will be reported in the future.

## Author contributions

All authors listed have made a substantial, direct, and intellectual contribution to the work and approved it for publication.

